# Associations of Polymorphisms in the Peroxisome Proliferator-Activated Receptor Gamma Coactivator-1 Alpha Gene With Subsequent Coronary Heart Disease: An Individual-Level Meta-Analysis

**DOI:** 10.3389/fphys.2022.909870

**Published:** 2022-06-23

**Authors:** Tessa Schillemans, Vinicius Tragante, Buamina Maitusong, Bruna Gigante, Sharon Cresci, Federica Laguzzi, Max Vikström, Mark Richards, Anna Pilbrow, Vicky Cameron, Luisa Foco, Robert N. Doughty, Pekka Kuukasjärvi, Hooman Allayee, Jaana A. Hartiala, W. H. Wilson Tang, Leo-Pekka Lyytikäinen, Kjell Nikus, Jari O. Laurikka, Sundararajan Srinivasan, Ify R. Mordi, Stella Trompet, Adriaan Kraaijeveld, Jessica van Setten, Crystel M. Gijsberts, Anke H. Maitland-van der Zee, Christoph H. Saely, Yan Gong, Julie A. Johnson, Rhonda M. Cooper-DeHoff, Carl J. Pepine, Gavino Casu, Andreas Leiherer, Heinz Drexel, Benjamin D. Horne, Sander W. van der Laan, Nicola Marziliano, Stanley L. Hazen, Juha Sinisalo, Mika Kähönen, Terho Lehtimäki, Chim C. Lang, Ralph Burkhardt, Markus Scholz, J. Wouter Jukema, Niclas Eriksson, Axel Åkerblom, Stefan James, Claes Held, Emil Hagström, John A. Spertus, Ale Algra, Ulf de Faire, Agneta Åkesson, Folkert W. Asselbergs, Riyaz S. Patel, Karin Leander

**Affiliations:** ^1^ Institute of Environmental Medicine, Karolinska Institutet, Stockholm, Sweden; ^2^ Division Heart and Lungs, Department of Cardiology, University Medical Center Utrecht, Utrecht University, Utrecht, Netherlands; ^3^ Division of Cardiovascular Medicine, Department of Medicine, Danderyd University Hospital, Karolinska Institutet, Stockholm, Sweden; ^4^ Department of Clinical Sciences, Danderyd University Hospital, Karolinska Institutet, Stockholm, Sweden; ^5^ Cardiovascular Division, John T. Milliken Department of Internal Medicine, Washington University School of Medicine, St. Louis, MO, United States; ^6^ Department of Medicine, Christchurch Heart Institute, University of Otago, Christchurch, New Zealand; ^7^ Cardiovascular Research Institute, National University of Singapore, Singapore, Singapore; ^8^ Institute for Biomedicine, Eurac Research, Bolzano, Italy; ^9^ Heart Health Research Group, The University of Auckland, Auckland, New Zealand; ^10^ Finnish Cardiovascular Research Center - Tampere, Department of Cardio-Thoracic Surgery, Faculty of Medicine and Health Technology, Tampere University, Tampere, Finland; ^11^ Department of Biochemistry and Molecular Medicine, Keck School of Medicine, University of Southern California, Los Angeles, CA, United States; ^12^ Department of Population and Public Health Sciences, Keck School of Medicine, University of Southern California, Los Angeles, CA, United States; ^13^ Department of Cardiovascular and Metabolic Sciences and Center for Microbiome and Human Health, Lerner Research Institute, Cleveland Clinic Ohio, Cleveland, OH, United States; ^14^ Department of Cardiovascular Medicine, Heart, Vascular and Thoracic Institute, Cleveland Clinic Ohio, Cleveland, OH, United States; ^15^ Department of Clinical Chemistry, Fimlab Laboratories Ltd., Tampere, Finland; ^16^ Finnish Cardiovascular Research Center - Tampere, Department of Clinical Chemistry, Faculty of Medicine and Health Technology, Tampere University, Tampere, Finland; ^17^ Finnish Cardiovascular Research Center - Tampere, Department of Cardiology, Faculty of Medicine and Health Technology, Tampere University, Tampere, Finland; ^18^ Heart Center, Department of Cardiology, Tampere University Hospital, Tampere, Finland; ^19^ Heart Center, Department of Thoracic Surgery, Tampere University Hospital, Tampere, Finland; ^20^ Division of Population Health and Genomics, School of Medicine, University of Dundee, Dundee, United Kingdom; ^21^ Division of Molecular and Clinical Medicine, School of Medicine, University of Dundee, Dundee, United Kingdom; ^22^ Department of Internal Medicine, Leiden University Medical Center, Leiden, Netherlands; ^23^ Section of Gerontology and Geriatrics, and Department of Cardiology, Leiden University Medical Center, Leiden, Netherlands; ^24^ Laboratory of Experimental Cardiology, University Medical Center Utrecht, Utrecht University, Utrecht, Netherlands; ^25^ Department of Cardiology, Radboud University Nijmegen Medical Centre, Nijmegen, Netherlands; ^26^ Amsterdam University Medical Centers, Department of Respiratory Medicine, University of Amsterdam, Amsterdam, Netherlands; ^27^ Vorarlberg Institute for Vascular Investigation and Treatment (VIVIT), Feldkirch, Austria; ^28^ Private University in the Principality of Liechtenstein, Triesen, Liechtenstein; ^29^ Academic Teaching Hospital Feldkirch, Feldkirch, Austria; ^30^ Center for Pharmacogenomics and Precision Medicine, Department of Pharmacotherapy and Translational Research, University of Florida, Gainesville, FL, United States; ^31^ Division of Cardiovascular Medicine, College of Medicine, University of Florida, Gainesville, FL, United States; ^32^ Azienda Ospedaliero Universitaria, Sassari, Italy; ^33^ Department of Medicine and Intensive Care, County Hospital Bregenz, Bregenz, Austria; ^34^ Intermountain Medical Center Heart Institute, Salt Lake City, UT, United States; ^35^ Division of Cardiovascular Medicine, Stanford University, Stanford, CA, United States; ^36^ Central Diagnostics Laboratory, Division Laboratories, Pharmacy, and Biomedical Genetics, University Medical Center Utrecht, Utrecht, Netherlands; ^37^ Medicine Laboratory Unit, ASST Rhodense (Rho-Milano), Lombardy, Italy; ^38^ Department of Medicine and Health Sciences, University of Molise, Campobasso, Italy; ^39^ Heart and Lung Center, Helsinki University Hospital, University of Helsinki, Helsinki, Finland; ^40^ Finnish Cardiovascular Research Center - Tampere, Department of Clinical Physiology, Faculty of Medicine and Health Technology, Department of Clinical Physiology, Tampere University, Tampere, Finland; ^41^ Institute of Clinical Chemistry and Laboratory Medicine, University Hospital Regensburg, Regensburg, Germany; ^42^ LIFE Research Center for Civilization Diseases, Leipzig University, Leipzig, Germany; ^43^ LIFE Research Center for Civilization Diseases, Institute for Medical Informatics, Statistics and Epidemiology, Leipzig University, Leipzig, Germany; ^44^ Department of Cardiology, Leiden University Medical Center, Leiden, Netherlands; ^45^ Netherlands Heart Institute, Utrecht, Netherlands; ^46^ Uppsala Clinical Research Center, Uppsala University, Uppsala, Sweden; ^47^ Department of Medical Sciences, Cardiology, Uppsala University, Uppsala, Sweden; ^48^ Saint Luke´s Mid America Heart Institute, University of Missouri-Kansas City, Kansas City, MO, United States; ^49^ Department of Neurology and Neurosurgery, Brain Centre Rudolf Magnus and Julius Center for Health Sciences and Primary Care, University Medical Center Utrecht, Utrecht, Netherlands; ^50^ Faculty of Population Health Sciences, Institute of Cardiovascular Science and Institute of Health Informatics, University College London, London, United Kingdom; ^51^ Bart’s Heart Centre, St Bartholomew’s Hospital, London, United Kingdom

**Keywords:** polymorphisms, *PPARGC1A*, meta-analysis, SNPs, coronary heart disease, cohort studies

## Abstract

**Background:** The knowledge of factors influencing disease progression in patients with established coronary heart disease (CHD) is still relatively limited. One potential pathway is related to peroxisome proliferator–activated receptor gamma coactivator-1 alpha (PPARGC1A), a transcription factor linked to energy metabolism which may play a role in the heart function. Thus, its associations with subsequent CHD events remain unclear. We aimed to investigate the effect of three different SNPs in the *PPARGC1A* gene on the risk of subsequent CHD in a population with established CHD.

**Methods:** We employed an individual-level meta-analysis using 23 studies from the GENetIcs of sUbSequent Coronary Heart Disease (GENIUS-CHD) consortium, which included participants (*n* = 80,900) with either acute coronary syndrome, stable CHD, or a mixture of both at baseline. Three variants in the *PPARGC1A* gene (rs8192678, G482S; rs7672915, intron 2; and rs3755863, T528T) were tested for their associations with subsequent events during the follow-up using a Cox proportional hazards model adjusted for age and sex. The primary outcome was subsequent CHD death or myocardial infarction (CHD death/myocardial infarction). Stratified analyses of the participant or study characteristics as well as additional analyses for secondary outcomes of specific cardiovascular disease diagnoses and all-cause death were also performed.

**Results:** Meta-analysis revealed no significant association between any of the three variants in the *PPARGC1A* gene and the primary outcome of CHD death/myocardial infarction among those with established CHD at baseline: rs8192678, hazard ratio (HR): 1.01, 95% confidence interval (CI) 0.98–1.05 and rs7672915, HR: 0.97, 95% CI 0.94–1.00; rs3755863, HR: 1.02, 95% CI 0.99–1.06. Similarly, no significant associations were observed for any of the secondary outcomes. The results from stratified analyses showed null results, except for significant inverse associations between rs7672915 (intron 2) and the primary outcome among 1) individuals aged ≥65, 2) individuals with renal impairment, and 3) antiplatelet users.

**Conclusion:** We found no clear associations between polymorphisms in the *PPARGC1A* gene and subsequent CHD events in patients with established CHD at baseline.

## Introduction

Coronary heart disease (CHD) is a multifactorial disease caused by a complex interplay between genetic, behavioral, and environmental factors, with atherosclerosis as the main underlying component (Tiret, 2002). Several processes important for atherosclerosis, such as lipid homeostasis (Zhang et al., 2004; Lin et al., 2005), endothelial function, and inflammation, are potentially modulated by the peroxisome proliferator–activated receptor gamma coactivator-1 alpha (PPARGC1A), encoded by the *PPARGC1A* gene (Kadlec et al., 2016). PPARGC1A co-activates several transcription factors involved in energy metabolism and oxidative stress including peroxisome proliferator–activated receptors (PPARs) and nuclear respiratory factors (NRFs) (Liang and Ward, 2006).

Animal studies have shown the evidence of PPARGC1A involvement in cardiac energy metabolism (Arany et al., 2005; Rowe et al., 2010) during development (Lai et al., 2008a) and aging (Whitehead et al., 2018). Furthermore, PPARGC1A is dysregulated in heart failure (Sihag et al., 2009; Oka et al., 2020) and plays a role in endothelial regulation (Craige et al., 2016), atherosclerotic lesions (Kadlec et al., 2016), and may be involved in endogenous protective mechanisms (i.e., ROS and mitochondrial biogenesis) (Chen et al., 2011). Human studies have shown associations between a non-synonymous coding variant single nucleotide polymorphism (SNP) in *PPARGC1A* (G482S; rs8192678) and metabolic outcomes (Vandenbeek et al., 2017) such as adiposity, insulin resistance (Franks et al., 2014), type 2 diabetes (T2D) (Ek et al., 2001), and hypertension (Andersen et al., 2005). Other SNPs in this gene are less studied in relation to cardiometabolic outcomes, but there are a number of associations reported, for example, between rs7672915 (intron variant) and left-ventricular diastolic function (Juang et al., 2010) and between rs3755863 (T528T) and waist circumference and cholesterol levels (Brito et al., 2009; Mirzaei et al., 2012). Although not found associated with CHD in genome-wide association studies (Peden et al., 2011; Schunkert et al., 2011; Deloukas et al., 2013; Nikpay et al., 2015; van der Harst and Verweij, 2018), *PPARGC1A* polymorphisms have been associated with the risk of the first-time CHD event and severity in candidate gene association studies (Zhang et al., 2008; Yongsakulchai et al., 2016; Maciejewska-Skrendo et al., 2019). CHD is increasingly described to be a chronic disease with a dynamic nature (Knuuti et al., 2019) and different genetic components could be involved in its progression in different phases. To the best of our knowledge, associations between PPARGC1A and subsequent CHD outcomes have not been investigated.

The GENetIcs of sUbSequent Coronary Heart Disease (GENIUS-CHD) consortium has been established to investigate the genetic determinants of disease progression, following an index CHD event, as many patients are living with CHD due to increased survival rates after a CHD event, and little is known about the risk factors influencing disease progression (Patel et al., 2019a). We conducted an individual-level meta-analysis using data from 23 cohort studies within the GENIUS-CHD consortium (Patel et al., 2019a) to investigate the effect of three different SNPs in the *PPARGC1A* gene on the risk of subsequent CHD. We could not analyze more than three SNPs due to restrictions within the consortium. The SNP rs8192678 was selected based on extensive previously reported associations with cardiometabolic phenotypes (Arya et al., 2004; Andersen et al., 2005; Barroso et al., 2006; Ridderstråle et al., 2006; Povel et al., 2010). We also included rs7672915 and rs3755863 because, although less studied, they have been suggested to be involved in myocardial metabolism or metabolic traits (Brito et al., 2009; Juang et al., 2010; Mirzaei et al., 2012). In addition, we examined a number of secondary cardiovascular disease endpoints and all-cause mortality, as well as the possible effect modification by age, sex, co-morbidities, and medication use.

## Methods

### The Consortium

The GENIUS-CHD consortium is an international collaboration, established in 2014 to investigate the impact of genetics on secondary CHD events (http://www.genius-chd.com/). Details about the consortium and inclusion criteria are published elsewhere (Patel et al., 2019a; Patel et al., 2019b). In brief, it mainly includes prospective cohort studies where participants with established CHD at baseline were followed for secondary CHD events. The cases are defined as those experiencing a subsequent CHD event. Participating studies received the local institutional review board approval and included patients who had/provided the informed consent at the time of enrollment.

### Inclusion and Exclusion Criteria

**FIGURE 1:**
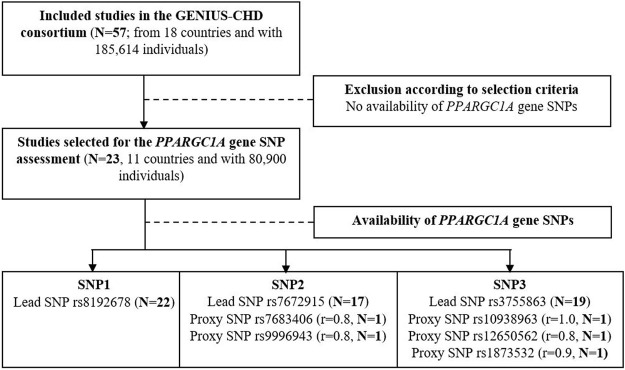
Flow chart of study selection criteria and available SNPs for the primary outcome. Correlation between lead and proxy SNP is indicated by r (source: LDproxy Tool; ldlink.nci.nih.gov in European).

Studies were included in the GENIUS-CHD consortium according to the following criteria: First, recruitment of participants with established CHD, defined as acute coronary syndrome or coronary artery disease (any revascularization procedures such as percutaneous coronary intervention, coronary bypass surgery, or a significant (50%) coronary artery plaque at angiography that affects any major epicardial vessel) at baseline or with a history thereof; second, availability of prospective follow-up and ascertainment of at least one clinical cardiovascular outcome (including all-cause mortality); and third, availability of samples, biomarkers, or *in silico* genotyping data. In the present study, we only included studies if SNP data in the *PPARGC1A* gene were available (Figure 1, flow-chart).

### Data Extraction and Quality Assessment

We examined three lead SNPs: rs8192678, rs7672915, and rs3755863. If those variants were not available, proxies in high linkage disequilibrium (*r* ^2^ > 0.8) were considered: rs7683406, rs9996943, rs1873532, rs10938963, and rs12650562 (Figure 1, flow-chart). All proxy SNPs are intronic variants.

The quality control of the genotype data was performed by each study prior to analysis. Minor allele frequencies (MAFs) and Hardy–Weinberg equilibrium (HWE) were examined by each study.

### Outcomes

The primary outcome was defined as myocardial infarction (MI) or CHD death during follow-up. Secondary outcomes were MI, coronary revascularization, heart failure, ischemic stroke, any stroke, any CVD (including MI, stroke, coronary revascularization, and CVD death), CHD death, CVD death, and all-cause death.

### Statistical Analysis

The associations between SNPs and cardiovascular outcomes were evaluated in individual studies assuming an additive genetic model and using time-to-event Cox proportional hazards models adjusted for age and sex. Analyses were performed using shared statistical scripts and harmonized datasets across the consortium (Patel et al., 2019a; Patel et al., 2019b).

The study-level effect estimates and their corresponding standard errors were entered in an inverse variance weighted fixed-effect meta-analysis model. The χ^2^ test for heterogeneity and the I^2^ statistic were used to quantify heterogeneity. Stratified analyses were performed for CHD subtypes at baseline: acute coronary syndrome (ACS) and coronary artery disease (CAD) with prior MI and CAD without prior MI. Stratification was also performed for the baseline patient-level characteristics of age (< or ≥65 years), sex, hypertension (physician-diagnosed or under treatment), T2D (physician-diagnosed or under treatment), body mass index (BMI) (18.5–24.9; 25–29.9; ≥30 kg/m2), statin use, antiplatelet use, renal impairment (eGFR<60 ml/kg/min), and left-ventricular impairment (left-ventricular ejection fraction<45% or diagnosed heart failure with impaired systolic function). Furthermore, sensitivity analyses were performed by stratifying two study-specific factors: European ancestry (a European study where >95% of the participants were of European ancestry versus non-European) and duration of the follow-up (< versus ≥5 years). In addition, we repeated the main analysis excluding cohorts departing from HWE (*p* < 0.05).

Effect sizes and confidence intervals (CI) were calculated using the two-sided α of 0.05, and results are presented as hazard ratios (HRs). Analyses at the coordinating centers were conducted by R software (version 3.4.1) (R Development Core Team), and the meta-analysis was performed using the EpiSheet tool (K. Rothman, www.krothman.org).

## Results

### Study Characteristics

In total, 23 studies from the GENIUS-CHD consortium with established CHD and available SNP data in the *PPARGC1A* gene were selected, with the lead SNPs available in 22, 17, and 19 studies and highly correlated (*r* ^2^ > 0.8 in Europeans) proxies available in 0, 2, and 2 studies for rs8192678 (non-synonymous variant G482S), rs7672915 (single nucleotide variant in intron 2), and rs3755863 (synonymous variant T528T), respectively (Figure 1). The participant characteristics and genotyping details (MAF and HWE p-value) of the SNPs under investigation are presented in Tables 1, 2.

**TABLE 1:** Characteristics of studies included in the meta-analysis.

Cohort	Study (country)	Design, CHD type	Year	Mean follow-up time, years (SD)	N recruited with CHD	Sex, % male	Mean age, years (SD)	European ancestry (%)	PubMed ID
AGNES	Arrhythmia Genetics in the Netherlands	Cohort, ACS	2001–2005	6.73 (4.75)	1,459	79.2	57.8 (10.7)	100	20622880
ANGNES	Angiography and Genes Study (Finland)	Cohort, mixed	2002–2005	8.20 (4.47)	588	65.5	64.1 (9.6)	100	21640993
CDCS	Coronary Disease Cohort Study (New Zealand)	Cohort, ACS	2002–2009	5.21 (2.15)	2,139	71.3	67.4 (12.0)	91.4	20400779
CTMM	CTMM Circulating Cells (Netherlands)	Cohort, mixed	2009–2011	0.97 (0.37)	713	69	62.6 (10.1)	96.5	23975238
FINCAVAS	Finnish Cardiovascular Study	Cohort, mixed	2001–2008	8.57 (3.99)	1,671	69.4	60.9 (11.0)	100	16515696
GoDARTSprevalent	Genetics of Diabetes Audit and Research in Tayside Scotland (I)	Population, CAD	2004–2012	3.47 (2.95)	1,261	61.1	71.3 (10.9)	99.8	29025058
GoDARTSincident	Genetics of Diabetes Audit and Research in Tayside Scotland (P)	Population, CAD	2004–2012	6.48 (3.06)	2,514	65.9	69.1 (9.4)	99.7	29025058
IATVB	Italian Atherosclerosis, Thrombosis and Vascular Biology Group	Cohort, ACS	1997–2006	10.47 (4.45)	1,741	90.8	40.0 (4.4)	100	21757122
LIFE-Heart	Leipzig (LIFE) Heart Study (Germany)	Cohort, mixed	2006–2014	1.62 (2.03)	5,564	77.2	63.9 (11.1)	100	32747942
LURIC	The Ludwigshafen Risk and Cardiovascular Health Study (Germany)	Cohort, mixed	1997–2000	8.58 (3.18)	2,320	76.6	63.8 (9.9)	100	11258203
OHGS	Ottawa Heart Genomics Study (Canada)	Cohort, mixed	2010–2013	1.77 (0.27)	546	73.8	65.6 (11.1)	100	NA
PLATO	The Study of Platelet Inhibition and Patient Outcomes (International)	RCT, ACS	2006–2008	0.86 (0.24)	18,624	69.5	62.6 (11.0)	98.3	19332184
PMI	Post Myocardial Infarction Study (New Zealand)	Cohort, ACS	1994–2001	8.56 (3.58)	1,057	78	62.8 (10.6)	91.1	12771003
PROSPER	Prospective Study of Pravastatin in the Elderly at Risk (Netherlands)	RCT, CAD	1997–1999	3.15 (0.71)	893	70.3	75.4 (3.4)	100	10569329
SHEEP	Stockholm Heart Epidemiology Program (Sweden)	Cohort, ACS	1992–1995	14.87 (5.91)	1,150	70.7	59.3 (7.2)	100	17667644
SMART	Second Manifestations of Arterial Disease (Netherlands)	Cohort, mixed	1999–2010	6.77 (3.86)	3,057	81.7	60.5 (9.3)	98.2	10468526
STABILITY	Stabilization of Atherosclerotic Plaque by Initiation of Darapladib Therapy trial (International)	RCT, CAD	2008–2010	3.60 (0.57)	10,786	82	64.7 (9.1)	86.1	24678955
UCP	Utrecht Cardiovascular Pharmacogenetic Study (Netherlands)	Cohort, mixed	1985–2010	8.00 (4.16)	1,508	75.4	64.1 (10.0)	100	25652526
UKB	United Kingdom Biobank (United Kingdom)	Population, CAD	2006–2010	6.39 (1.72)	12,045	80.6	69.9 (6.1)	94.2	1001779
VIVIT	Vorarlberg Institute for Vascular Investigation and Treatment Study (Austria)	Cohort, CAD	1999–2008	7.43 (2.91)	1,447	72	64.5 (10.5)	99.8	24265174
GENEBANK	Cleveland Clinic Genebank Study (United States)	Cohort, mixed	2001–2007	3.00 (0.00)	2,345	74.3	61.5 (11.1)	100	21475195
INVEST	International Verapamil SR Trandolopril Study Genetic Substudy INVEST-GENES (United States/International)	RCT, CAD	1997–2003	2.83 (0.82)	5,979	44	66.1 (9.7)	38.0	21372283, 17700361
UCORBIO	Utrecht Coronary Biobank (Netherlands)	Cohort, mixed	2011–2014	1.6 (0.9)	1,493	75.6	65.4 (10.3)	72.4	NA
Additional studies not included in primary outcome analysis but included in secondary outcome analyses of all-cause mortality.									
COROGENE	Corogene Study (Finland)	Cohort, ACS	2006–2008	7.7 (0.5)	1,489	70.9	64.7 (11.9)	100	21642350
MDCS	Malmo Diet and Cancer Study (Sweden)	Population, CAD	1991–1996	8.3 (8.0)	4,546	60.2	58.0 (7.6)	100	19936945
TRIUMPH	Translational Research Investigating Underlying Disparities in Acute Myocardial Infarction Patient’s Health Status (United States)	Cohort, ACS	2005–2008	0.97 (0.15)	2,062	72.2	59.8 (12.1)	100	21772003
WTCCC (BHF)	WTCCC CAD Study (United Kingdom)	Cohort, mixed	1998–2003	10.05 (2.81)	1,926	79.3	60.0 (8.1)	100	16380912, 17634449

More detailed information is available in Reference number 28: Patel RS,et al. (2019) Circ Genom Precic Med.

CHD, coronary heart disease; ACS, acute coronary syndrome; CAD, coronary artery disease; RCT, randomized controlled trial; SD, standard deviation.

**TABLE 2:** Minor allele frequencies (MAFs) and p-values for Hardy–Weinberg equilibrium (P_HWE_) for the three SNPs in the studies included in the meta-analysis.

Cohort	MAF rs8192678 (G482S)	PHWE rs8192678 (G482S)	MAF rs7672915 (intron 2)	PHWE rs7672915 (intron 2)	MAF rs3755863 (T528T)	PHWE rs3755863 (T528T)
AGNES	0.331	0.106	0.404^a^	0.842^a^	0.395	0.550
ANGNES	0.316	0.075	0.385	0.265	0.343	0.280
CDCS	0.345	0.880	0.465	0.254	0.402	0.741
CTMM	0.345	0.720	0.430	0.242	0.402	0.237
FINCAVAS	0.320	0.261	0.355	0.0006^b^	0.350	0.162
GoDARTSprevalent	0.348	0.784	0.466	0.485	0.409	0.572
GoDARTSincident	0.325	0.523	0.444	0.064	0.382	0.416
IATVB	0.360	0.909	-	-	0.441^a^	0.672^a^
LIFE-Heart	0.323	0.135	0.432	0.950	0.367	0.109
LURIC	0.345	0.776	0.448	0.571	0.395	0.654
OHGS	0.287	0.806	0.424^a^	0.215^a^	0.355	0.097
PLATO	0.325	0.698	0.451	0.374	0.374	0.701
PMI	0.348	0.015^b^	0.435	0.093	0.404	0.032
PROSPER	0.338	0.582	0.442	0.510	0.399	0.420
SHEEP	0.339	0.946	0.399	0.804	0.391	0.416
SMART	0.339	0.965	-	-	-	-
STABILITY	0.334	0.778	0.463	0.026^b^	0.385	0.714
UCP	0.353	0.734	0.435	0.434	0.411^a^	1.0^a^
UKB	0.342	0.982	0.061	0.909	0.005	0.846
VIVIT	-	-	-	-	0.477^a^	0.270^a^
GENEBANK	0.348	0.716	0.423	0.472	0.4	0.931
INVEST	0.301	0.034^b^	0.498	0.554	0.374	0.385
UCORBIO	0.337	0.643	-	-	-	-
Additional studies are not included in primary outcome analysis but included in secondary outcome analyses of all-cause mortality.						
COROGENE	0.322	0.373	0.333	0.162	0.350	0.609
MDCS	0.342	0.325	0.379	0.536	0.392	0.769
TRIUMPH	0.370	0.002^b^	0.464	0.103	0.404	0.015^b^
WTCCC (BHF)	0.333	0.341	0.460	1.0	0.408	1.0

a Indicates the use of a highly correlated proxy (AGNES, rs9996943; OHGS, rs7683406; IATVB, rs10938963; UCP, rs1873532; and VIVIT, rs12650562).

b Studies with p H_WE_<0.05 were excluded in sensitivity analyses for the primary outcome.

### Meta-Analysis Results

**FIGURE 2:**
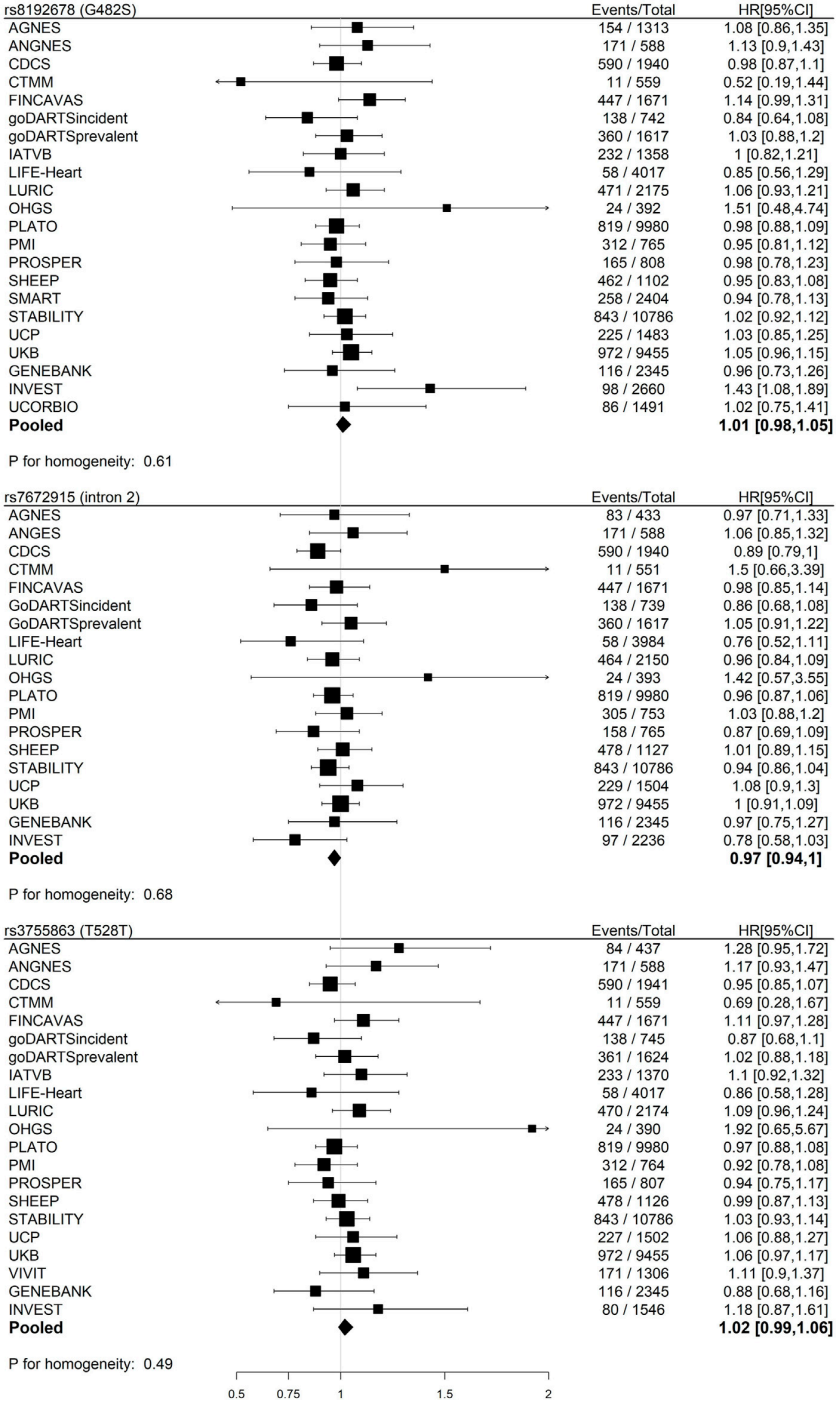
Meta-analyses of the associations between three SNPs in the *PPARGC1A* gene and primary outcome (CHD death or myocardial infarction) in participants with baseline CHD within GENIUS-CHD using an additive, fixed-effect model adjusted for age and sex.

**FIGURE 3:**
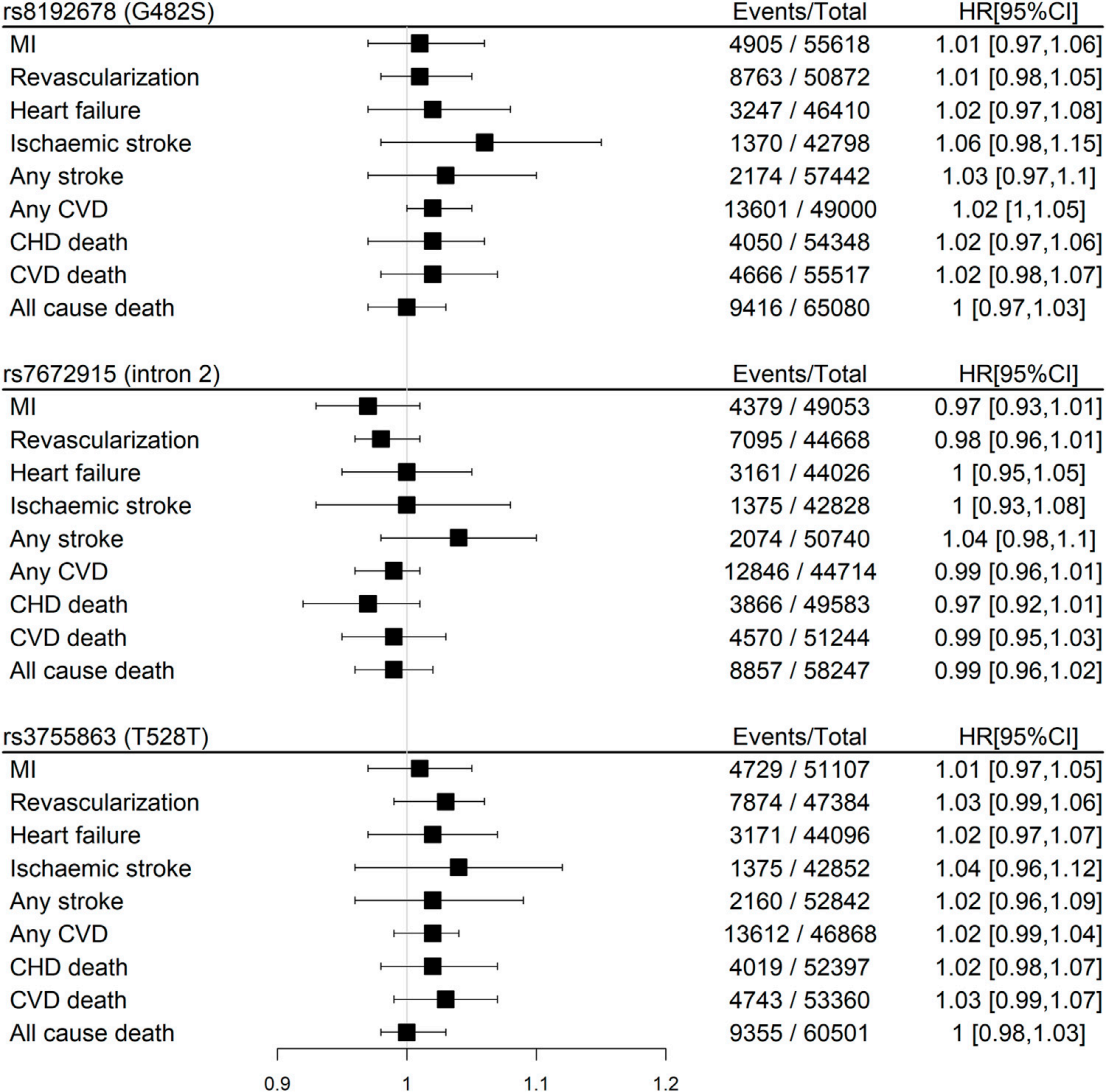
Meta-analyses pooled results of the associations between three SNPs in the *PPARGC1A* gene and secondary outcomes in participants with baseline CHD within GENIUS-CHD using an additive, fixed-effect model stratified for age and sex (p_homogeneity_>0.05 for all outcomes). Abbreviations: CHD, coronary heart disease; CVD, cardiovascular disease; MI, myocardial infarction.

We found null associations for all three SNPs with the primary outcome of CHD death or MI (Figure 2). Similarly, null associations were found for all three SNPs with all secondary outcomes (Figure 3).

### Stratified Meta-Analysis

**FIGURE 4:**
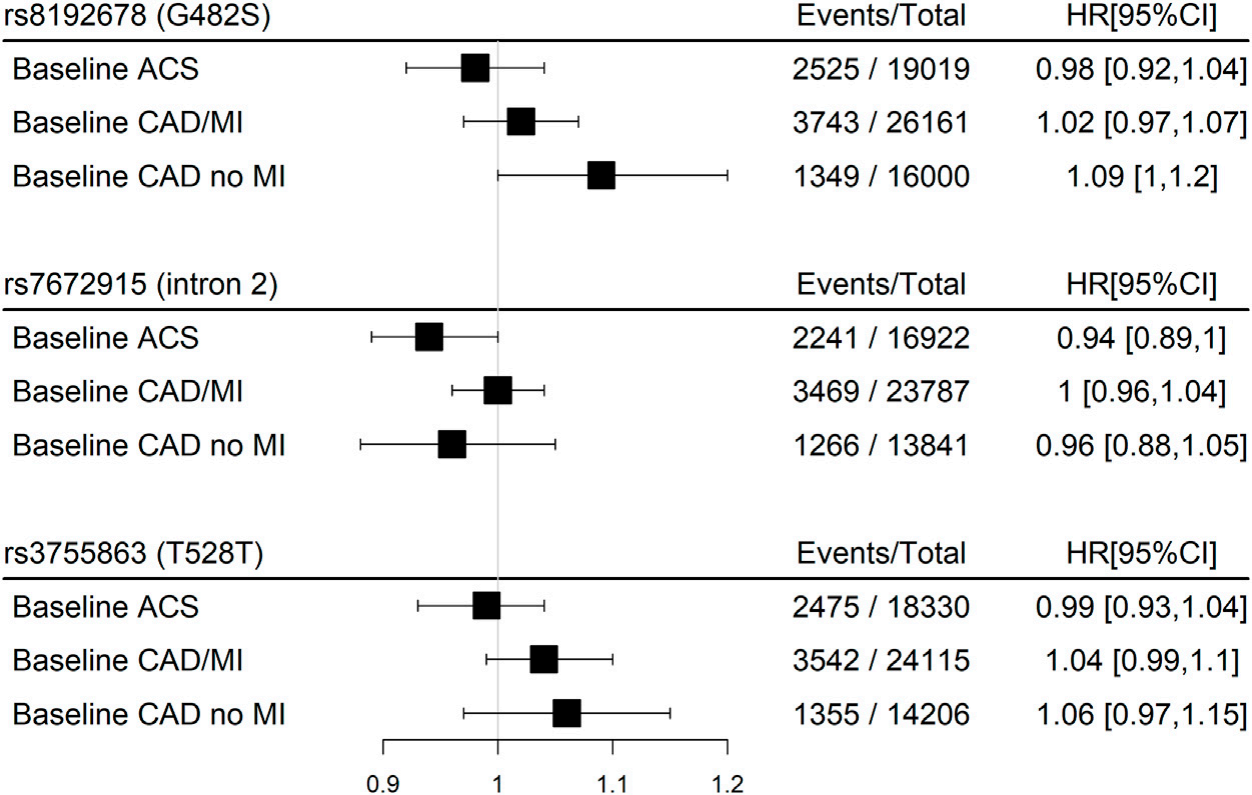
Meta-analyses pooled results of the associations between three SNPs in the *PPARGC1A* gene and the main outcome (CHD death or myocardial infarction) in participants with baseline CHD within GENIUS-CHD using an additive, fixed-effect model stratified for the baseline CHD subtype (p_homogeneity_>0.05 for all outcomes).Abbreviations: ACS, Acute Coronary Syndrome; CAD, Coronary Artery Disease; MI, Myocardial Infarction.

**FIGURE 5:**
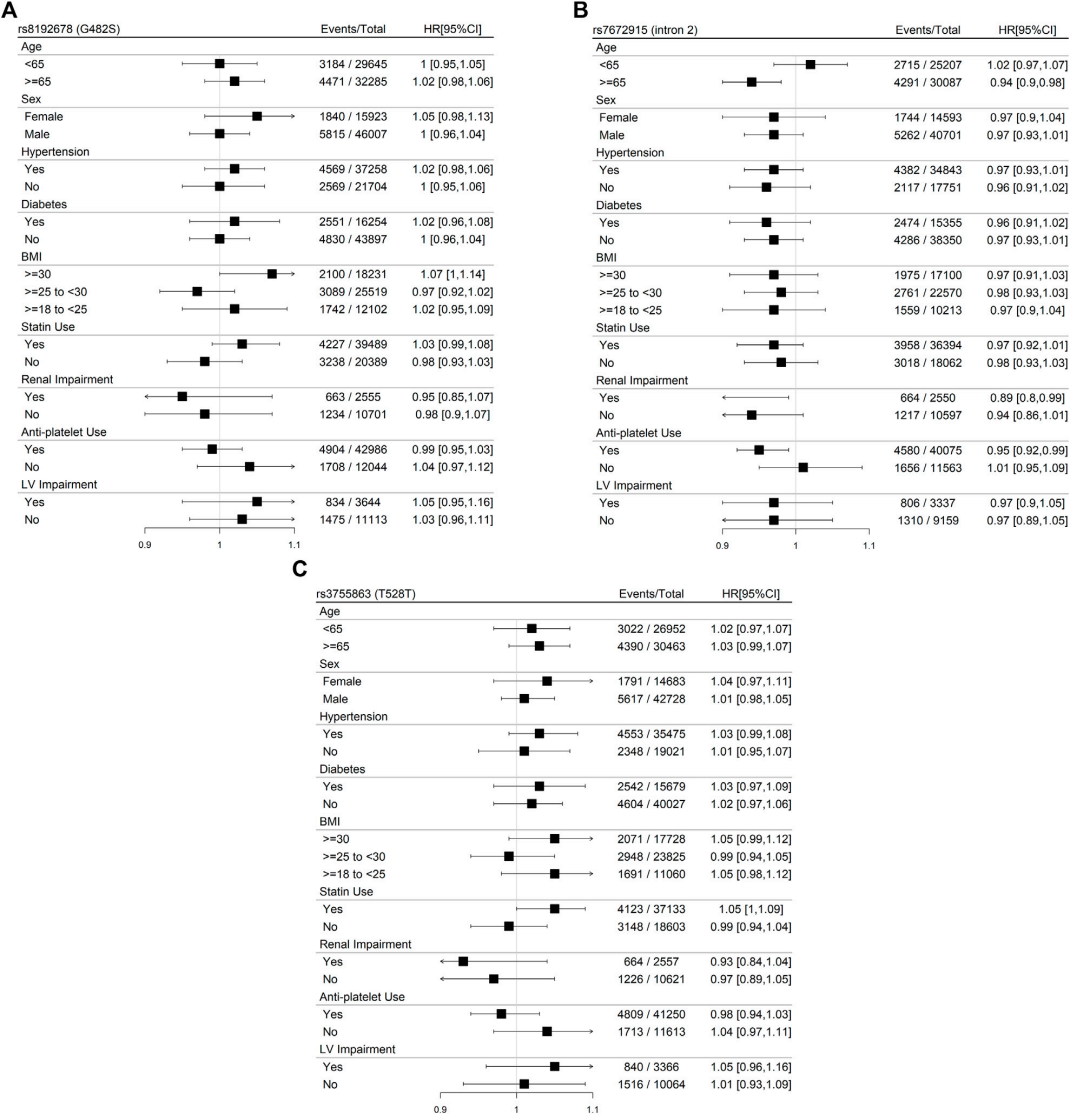
Panel **(A):** Meta-analyses of the associations between rs8192673 (G482S) in the *PPARGC1A* gene and the primary outcome (CHD death or myocardial infarction) in participants with baseline CHD within GENIUS-CHD using an additive, fixed-effect model stratified for patient-level characteristics. LV, left ventricular. Panel **(B):** Meta-analyses of the associations between rs7672915 (intron 2) in the *PPARGC1A* gene and the primary outcome (CHD death or myocardial infarction) in participants with baseline CHD within GENIUS-CHD using an additive, fixed-effect model stratified for patient-level characteristics. LV, left ventricular. Panel **(C):** Meta-analyses of the associations between rs3755863 (T528T) in the *PPARGC1A* gene and the primary outcome (CHD death or myocardial infarction) in participants with baseline CHD within GENIUS-CHD using an additive, fixed-effect model stratified for patient-level characteristics. LV, left ventricular.

Stratification by the CHD subtype at baseline resulted in borderline significant direct associations for rs8192678 (G482S) with the primary outcome for baseline CAD without MI and borderline inverse associations for rs7672915 (intron 2) for baseline ACS (Figure 4). A significant inverse association was found for rs7672915 (intron 2) with the primary outcome among the ≥65 years of age category as well as for renal impairment and antiplatelet use (Figure 5B). There were no significant associations between any of the SNPs and the primary outcome in the models stratified by sex, hypertension, T2D, BMI, statin use, and left-ventricular impairment (Figures 5A–C).

Sensitivity analyses only indicated marginal differences. Neither exclusion of cohorts deviating from HWE nor stratification by European ancestry changed associations with the primary outcome (data not shown). Stratification by follow-up time also resulted in null associations, except for significant inverse associations for rs7672915 (intron 2) and the primary outcome in the stratum with follow-up <5 years (HR: 0.93, 95% CI 0.88–0.99).

## Discussion

This meta-analysis resulted in overall null associations between three polymorphisms in the *PPARGC1A* gene studied in relation to the risk of subsequent CHD events (primary outcome) in a population with established CHD. The polymorphism rs7672915 (intron 2) was, however, observed to be borderline inversely associated with subsequent CHD events.

The results were generally consistent across the strata of CHD subtypes at baseline as well as of patient- and study-level characteristics. However, inverse associations with the primary outcome were seen for rs7672915 (intron 2) amongst those with age ≥65, renal impairment, and use of antiplatelets. In addition, inverse associations with the primary outcome were seen for rs7672915 (intron 2) among studies having a follow-up <5 years.

### Human Studies on Polymorphisms in the *PPARGC1A* Gene

Our results suggested that the *PPARGC1A* gene in patients with established CHD does not play an important role in disease progression, leading to subsequent CHD events. Previous research study has not investigated this relationship. However, there are studies that indicate that the *PPARGC1A* gene is important for the development of CAD (Zhang et al., 2008; Yongsakulchai et al., 2016; Maciejewska-Skrendo et al., 2019) and cardiometabolic disease phenotypes (Ek et al., 2001; Barroso et al., 2006; Xie et al., 2007; Lai et al., 2008b; Vimaleswaran et al., 2008; Yang et al., 2011; Franks et al., 2014; Jemaa et al., 2015; Kruzliak et al., 2015). The *PPARGC1A* gene (rs8192678; G482S) was associated with an increased risk of CAD in a Chinese population (Zhang et al., 2008). Moreover, the *PPARGC1A* gene (rs8192678; G482S), alone as well as in combination with polymorphisms in *PPARG* and liver X receptor α (*LXRA*), associated with an increased risk and severity of CAD in a Thai population (Yongsakulchai et al., 2016). The *PPARGC1A* gene (rs8192678; G482S) was further associated with T2D in a Tunisian population (Jemaa et al., 2015), with waist circumference among Slovenian participants with T2D (Kruzliak et al., 2015) and with severe hypertension in a Chinese population (Xie et al., 2007). The *PPARGC1A* gene was also identified in a search for protein-level interactions with transcripts mapped nearest to T2D susceptibility loci (Morris et al., 2012). Although the rs8192678; G482S Ser482 allele appears to be associated with increased obesity and T2D susceptibility (Vandenbeek et al., 2017) as well as a poorer therapeutic efficacy of rosiglitazone (Zhang et al., 2010), its carriers also appear to respond better to caloric restriction (Goyenechea et al., 2008) and bariatric surgery (Geloneze et al., 2012). In the Boston Puerto Rican Health Study, they found associations between polymorphisms in the *PPARGC1A* gene and DNA damage, T2D, and CVD (Lai et al., 2008b). However, meta-analysis only indicated modest roles within specific ethnicity and age groups for polymorphisms in the *PPARGC1A* gene (rs8192678; G482S) with T2D (Barroso et al., 2006; Yang et al., 2011) and hypertension (Vimaleswaran et al., 2008). The Ser482 allele carried an increased risk for hypertrophic cardiomyopathy in a community-based cross-sectional study in China (Wang et al., 2007). However, another study in a Russian population did not find evidence for such an association (Nikitin et al., 2010). The two other SNPs under investigation were less studied but have been shown to associate with metabolic traits (Brito et al., 2009; Juang et al., 2010; Mirzaei et al., 2012): rs7672915 (intron 2) associated with left-ventricular diastolic function in Caucasians (Juang et al., 2010) whereas rs3755863 (T528T) was found to be associated with waist circumference in European children (Brito et al., 2009) and with LDL cholesterol in an adult population consisting mostly of obese women (Mirzaei et al., 2012). Furthermore, rs3755863 (T528T) seemed to decrease *PPARGC1A* expression levels in cellular models (Mirzaei et al., 2012). Although results from subgroup analyses always should be interpreted with caution, it is possible that the significant association between rs7672915 (intron 2) and the primary outcome seen only in the subgroup of individuals aged 65 years or older in our study population may be relevant; age-related risk factors could interact with genetics and increase vulnerability to subsequent events. Based on similar reasoning, the significant associations we observed in subgroups with impaired renal function and users of antiplatelets, respectively, could be relevant. Also, the fact that rs7672915 (intron 2) was significantly associated with our primary outcome when the basis for the analysis was limited to including cohort data with less than 5 years of follow-up may indicate that the *PPARGC1A* gene plays a role in CHD progression, possibly in repair and recovery after an initial event in the short term.

### Mechanistic Studies on *PPARGC1A* and Cardiometabolic Health

The *PPARGC1A* gene, located on chromosome 4, encodes for a protein consisting of 798 amino acids in humans. It is highly expressed in tissues abundant in mitochondria such as the liver (in fasting states) (Yoon et al., 2001), kidney, brown adipose tissue, skeletal muscle, brain, and heart (Puigserver et al., 1998). PPARGC1A activates transcription factors by inducing a conformational change after binding to them, which increases the affinity of the transcription complex to other coactivators that have histone acetyltransferase activity. This increased affinity will then lead to the acetylation of histone proteins and conformational alterations that allow the increased accessibility of DNA to the transcription complex (Puigserver et al., 1999). Several pathways involving *PPARGC1A* and energy metabolism have been described, for example, mitochondria biogenesis, glucose/fatty acid metabolism, remodeling of fiber muscle composition, and adaptive thermogenesis (Liang and Ward, 2006). The effect of PPARGC1A on the mitochondrial metabolism, especially, can have implications for cardiac health by regulating the fuel availability and the amount of reactive oxygen species in the heart (Di et al., 2018). PPARGC1A target genes that are thought to play a role in cardiac health are estrogen-related receptors (ERRs; i.e., ERRα, ERRβ, and ERRγ) and nuclear respiratory factor-1, which activate many mitochondrial genes, as well as PPARs (i.e., PPARα, PPARβ, PPARγ, and PPARδ), which play important roles in the fatty acid uptake and oxidation in the heart (Di et al., 2018). Any dysregulation in *PPARGC1A* may be detrimental; studies have shown that its downregulation increased vascular stress (Kadlec et al., 2016), oxidative stress and inflammation (Waldman et al., 2018; Rius-Pérez et al., 2020), impaired mitochondrial function, and reduced antiapoptotic and angiogenic responses (Mahmood et al., 2019), whereas its upregulation induced pathological changes in mitochondrial biogenesis, contributing to cardiac disease (Lehman et al., 2000; Le Chen and Knowlton, 2011; Caravia et al., 2018). One of the SNPs (rs8192678; G482S) was recently shown to decrease the stability, impact structural conformation, and catalytic function of the PPARGC1A protein, which could be detrimental for CAD (Taghvaei et al., 2021). However, this is not reflected in our findings of null associations for the three tested SNPs in the *PPARGC1A* gene with subsequent CHD events. Our findings of inverse associations of rs7672915, intron 2 in subgroups (older age, with renal impairment, and antiplatelet users) could be random findings or due to the role of PPARGC1A in mitochondria subsequently affecting the aging process (Wenz, 2011), kidney disease (Lynch et al., 2018), and platelet function (Melchinger et al., 2019).

### Limitations and Strengths

There are several limitations which may have attenuated or diluted the effect estimates. First, when studying cohorts of patients, as in this study, there is always a possibility that the index event bias may influence the results (Dahabreh and Kent, 2011; Patel et al., 2019a). Our study population consists of CHD survivors subjected to varying types of preventive actions, including lifestyle changes and drug treatments, which may have impacted risks for recurrent events and death. In addition, there is a possibility that individuals who died early with the disease have a more severe phenotype and that the degree of severity is linked to the presence of the genetic variants we studied. However, in the previously published genome-wide studies of genetic variants in relation to the risk of first-time events of CHD, the current genetic variants were not included among the significant association findings (Peden et al., 2011; Schunkert et al., 2011; Deloukas et al., 2013; Nikpay et al., 2015; van der Harst and Verweij, 2018). Furthermore, if the index event is a consequence of a strong risk factor, there may be lower levels of exposure to other—individually weaker—independent risk factors in the selected population, which could have attenuated associations between genetic variants and the risk of subsequent events in our study (Patel et al., 2019b). Second, we had no information on the age of onset of the index CHD event or on whether revascularization procedures were late-staged (belonging to the index event) or unplanned and symptom-driven (true secondary event). Third, the variability in the follow-up between studies could impact the findings through outcome misclassification. Fourth, it is possible that other SNPs, outside our selected three, in the *PPARGC1A* gene play a key role. Finally, in the present study, we only investigated single SNP associations within a single gene whereas it could be relevant to also address gene–gene interactions and polygenetic scores. The major strength of this study, however, is the large number of studies and individuals included. This allowed us to make reasonably conclusive inferences.

## Conclusion

The findings from this large individual-level meta-analysis do not indicate the involvement of the *PPARGC1A* gene in the progression to secondary CHD events amongst people who experienced an index CHD event. However, future research studies on the potential role of *PPARGC1A* in subgroups of patients with established CHD, in relation to the risk of recurrence, may be warranted.

## Data Availability

The data analyzed in this study are subjected to the following licenses/restrictions: Individual participant-level data for each participating study were not collected for the present project and will, therefore, not be made available. Further details and contact information are available at http://krothman.hostbyet2.com/. Requests to access these datasets should be directed to www.genius-chd.org.
